# A hierarchical dynamic Bayesian learning network for EMG-based early prediction of voluntary movement intention

**DOI:** 10.1038/s41598-023-30716-7

**Published:** 2023-03-23

**Authors:** Yongming Chen, Haihong Zhang, Chuanchu Wang, Kai Keng Ang, Soon Huat Ng, Huiwen Jin, Zhiping Lin

**Affiliations:** 1 Institute for Infocomm Research, Agency for Science, Technology and Research, Singapore, Singapore; 2 School of Electrical and Electronic Engineering, Nanyang Technological University, Singapore, Singapore; 3 School of Computer Science and Engineering, Nanyang Technological University, Singapore, Singapore

**Keywords:** Biomedical engineering, Electrical and electronic engineering

## Abstract

Decoding human action intention prior to motion onset with surface electromyograms (sEMG) is an emerging neuroengineering topic with interesting clinical applications such as intelligent control of powered prosthesis/exoskeleton devices. Despite extensive prior works in the related fields, it remains a technical challenge due to considerable variability of complex multi-muscle activation patterns in terms of volatile spatio-temporal characteristics. To address this issue, we first hypothesize that the inherent variability of the *idle* state immediately preceding the motion initiation needs to be addressed explicitly. We therefore design a hierarchical dynamic Bayesian learning network model that integrates an array of Gaussian mixture model – hidden Markov models (GMM-HMMs), where each GMM-HMM learns the multi-sEMG processes either during the idle state, or during the motion initiation phase of a particular motion task. To test the hypothesis and evaluate the new learning network, we design and build a upper-limb sEMG-joystick motion study system, and collect data from 11 healthy volunteers. The data collection protocol adapted from the psychomotor vigilance task includes repeated and randomized binary hand motion tasks (push or pull) starting from either of two designated *idle* states: relaxed (with minimal muscle tones), or prepared (with muscle tones). We run a series of cross-validation tests to examine the performance of the method in comparison with the conventional techniques. The results suggest that the idle state recognition favors the dynamic Bayesian model over a static classification model. The results also show a statistically significant improvement in motion prediction accuracy by the proposed method (93.83±6.41%) in comparison with the conventional GMM-HMM method (89.71±8.98%) that does not explicitly account for the idle state. Moreover, we examine the progress of prediction accuracy over the course of motion initiation and identify the important hidden states that warrant future research.

## Introduction

By probing muscle activation non-invasively, surface Electromyography (sEMG) has become a prominent neuroengineering tool for detecting voluntary upper limb motion intention, and accounts for approximately 40% of the motion intention detection strategies related to robotic upper-limb orthoses according to a recent review^1^. The technology of sEMG-based motion intention detection will enable intelligent user-driven functional movement training with the emerging wearable hand robot technology, while such training has demonstrated values for rehabilitation and assistive utilities in stroke survivors^2^. Other applications such as upper-limb prosthetic control^3–5^ and rehabilitation exoskeleton^6,7^ further illustrate its clinical value for patients with impaired mobility. This applied research discipline has seen a steady pace of acceleration in recent years thanks to the advent of low-cost high-user-compliance sensing solutions such as the Myo armband^8^.

A large variety of detection algorithms have been reported that broadly fall into two categories. In the first category, the algorithms typically employ a time window and reduce the detection problem into classification of sEMG in the individual time windows. In the second, the algorithms seek to capture the complex dynamics of sEMG activities continuously.

The first class of algorithms can be referred to as ”static” models^9^, which often examine quasi-stationary sEMG patterns over a short time window typically of a few hundred milliseconds. For example, a forearm sEMG system^10^ used a 250ms time window and a parallel conditional classifier and achieved average classification accuracy rate of 6.6% for discrete (1 DOF) hand and wrist movement and 10.9% for combined movements (2 DOF). In another study^11^, a sliding window with a 256ms length and a 32ms step size was used with a Gaussian mixture model (GMM) classifier to recognize 6 upper-limb motions. Similar methods but using different pattern recognition techniques have also been reported, including K-Nearest-Neighbor (k-NN)^12^, Support Vector Machine (SVM)^13^, Linear Discriminant Analysis (LDA)^14^, and Extreme Learning Machines (ELM)^15^ etc. However, the static model approach may face inherent limitations associated with either fixed or variable window length and difficulties in capturing time dependency patterns of importance^9^.

The second category of algorithms explore important temporal information associated with the inherently non-stationary sEMG over longer or indefinite time ranges. By capturing the temporal dependencies therein, these models may recognize inherent temporal structures of sEMG activation patterns^16–18^. Especially, a typical dynamic Bayesian network model called the hidden Markov model (HMM) has widely been applied to gesture recognition^19^. In a recent study, for example, a natural Bayesian nonparametric extension of the classical HMM was applied to recognize 17 gesture tasks^18^. It has also been demonstrated that a HMM model of sEMG can be used to estimate the elbow joint angle during the flexion/extension motion^20^.

While these existing dynamic models usually consider classifying motion during the process of the motion or even at the end of it, other researchers have also explored predicting specific motion parameters in advance. For example, a deep-learning method was introduced to predict the knee angle at the next 100ms during walking^21^. It has also been demonstrated that sEMG and kinematics data may be combined to predict the head motion at the next 17.5ms, although the result has yet to demonstrate significant value of sEMG on top of kinematics for the prediction^22^.

Comparing with the above research works, early prediction of motion intention during motion initiation has received much less attention so far, whilst early prediction is highly wanted in various rehabilitation and assistive applications. As suggested by an empirical study^23^, in order to avoid any noticeable latency in prosthesis control, the motion intention should be predicted no later than 100 to 150ms after the onset of sEMG activation. In another independent study^24^, the authors have shown that Gaussian mixture models can predict upper-limb Go-forward or Go-backward motion at 100ms before the actual move begins.

Prediction of motion intention during motion initiation (e.g. before motion onset) requires learning the sEMG processes over both the *idle* state and the motion initiation process. However, to the best of our knowledge, the complexity and variability of these sEMG processes have not been sufficiently accounted for. In contrast to existing approaches, we propose that the *idle* state preceding the motion initiation process should be addressed explicitly, since the variability of this state will determine the process of how the skeletal-muscular system changes from the unintentional state of *idle* to activating the related multiple muscle groups individually.

Therefore, this proof-of-concept research aims to demonstrate the feasibility of designing machine learning that explicitly accounts for both the variability of the *idle* state and the uncertainty of the temporal processes of motion initiation. The research also needs to empirically examine our hypothesis that the new design can lead to higher accuracy in motion intention prediction.

Particularly, we design a hierarchical machine learning method that comprises a set of dynamic Bayesian networks (implemented with Gaussian mixture model – hidden Markov models, or GMM-HMMs). Each of them either learns sEMG data in a particular class of the *idle* state, or learns sEMG activity processes during motion initiation for any particular combination of the motion class and the idle state. Thanks to the Bayesian methodology, this method allows to compute, at any arbitrary time point before motion onset, the likelihood of the upcoming motion belonging to each motion class of interest.

To empirically validate the new machine learning method, we design a data collection protocol to acquire upper limb sEMG recordings associated with different hand movements as well as different idle states. We seek ethical approval and recruit 11 participants in this proof-of-concept study. Various tests are employed to examine the new machine learning design in comparison with the conventional method.

The rest of the paper is organized as follows. Section 2 describes the data collection system and procedure, followed by the proposed machine learning model and prediction algorithms. Section 3 reports the offline tests and the empirical results. Section 4 presents discussions and our conclusion.

## Materials and methods

This section consists of the methodology of the data acquisition and the proposed hierarchical dynamic Bayesian learning network. We first introduce the data collection system and the upper-limb voluntary movement experimental paradigm to collect the sEMG signals during a binary upper-limb motion task in two separate idle states. The hierarchical architecture of the machine learning model and the motion intention prediction algorithm are then introduced. Lastly, we provide a detailed introduction to the algorithm of the feature extraction and the Gaussian mixture model- hidden Markov model (GMM-HMM).

### Participants and sEMG collection procedure

Human sEMG data collection was conducted with 11 healthy volunteers who gave written informed consents in advance. The study was performed in accordance with the protocol IRB Ref. No. 2020-006, which was approved beforehand by the ethics review board of the A*STAR Human Biomedical Research (HBR) office. The research followed all relevant guidelines/regulations. While anthropometric characteristics may affect the quality of sEMG, this protocol was not particularly designed to investigate these characteristics. Nevertheless, our participants were all within the normal range of Body Mass Index.

The data collection system consisted of an integrated sEMG-hand-motion measurement device including a bio-potential amplifier (Neuroscan NuAmps) for sEMG measurement and a modified Logitech Extreme3D joystick for hand-motion measurement. Both the sEMG and the joystick measurements operated at the sampling frequency of 500Hz, while the hardware stimcode mechanism on the amplifier was used to precisely determine the sampling times of the received time samples. The recorded sEMG signals were high-pass filtered at 15Hz cutoff frequency by using a third order Butterworth high pass filter and notch filtered (50Hz) by using a third order Butterworth notch filter to remove unwanted noises and interference.

**Figure 1:**
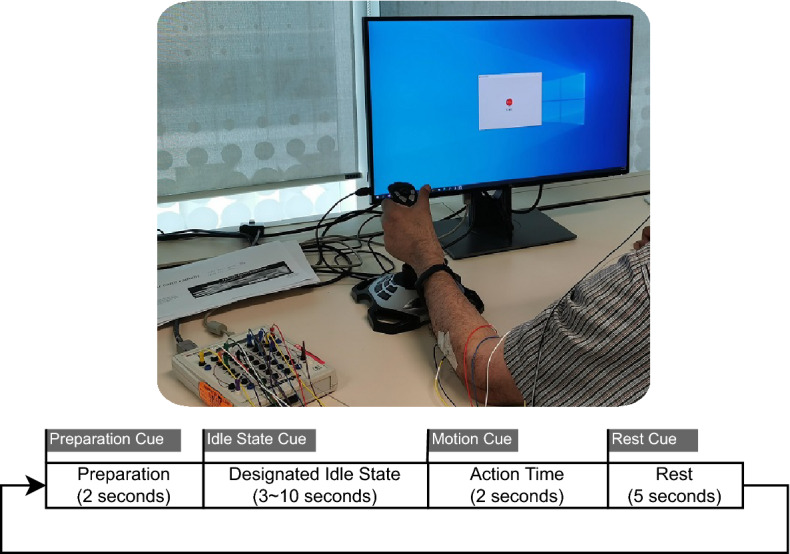
The data collection system and the time structure of a motion task trial.

The data collection session for each human subject consisted of a sequence of randomised and computer-guided hand motion tasks. Only one hand was considered for the motion tasks and the particular side was chosen according to individual preference. The system and the time structure of the task is illustrated in Fig. 1. Following the style of the well-established psychomotor vigilance task (PVT)^25^ paradigm, it used visual task cues on the computer display to designate which of the three tasks the user needs to perform as quickly as possible; hand pushing to the opposite side (hereafter *push*); hand pulling to the same side (hereafter *pull*); and thumb pressing the control button on the joystick. Note that the button press motion data were excluded from this research so that we should focus on binary-class motion prediction involving *push* and *pull* tasks only.

**Figure 2:**
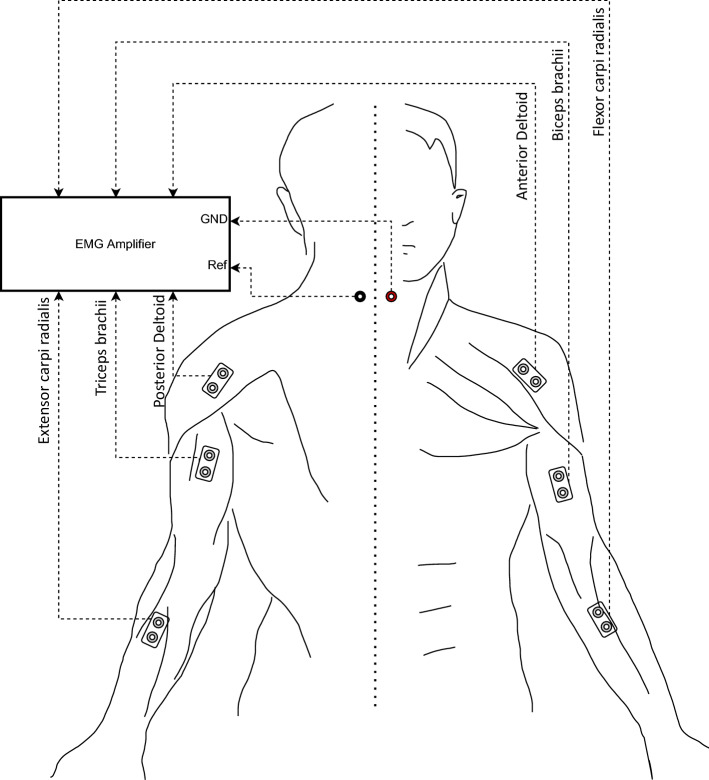
The configuration of the sEMG measurement system. Notice the left/right side depicts the perspective from the back/front.

In relevance to the hand motion tasks, six muscle groups were selected for bipolar sEMG measurement, namely triceps, biceps, anterior deltoid, posterior deltoid, flexor carpi radialis, and extensor carpi radialis. See Fig. 2 for the illustration of the sensor positions as well as the ground and reference electrode positions.

The total number of *push* and pull task repetitions for each human subject is 120, which is equally shared by the two motion tasks. The sequence of the tasks was randomized such that the user would not be able to anticipate the upcoming task, hence minimizing unwanted involuntary/voluntary activation of muscles. To further minimize possibly compounding neuromuscular factors related to unwanted motion planning and initiation that should not be present in the designated *idle* state, our computer programme also randomized the time interval (uniformly between 3 and 10 seconds) from the beginning of the designated idle time till the task cue onset. Additionally, we instructed the subjects to minimize any motion intention or thoughts related to the hand until the task cue was given. We believe these design elements would combine to create a clean state of *idle* free of motion intention related to the upperlimb.

Another new feature of the protocol design relates to the aforementioned hypothesis about the variability of the *idle* state. Specifically, we designated two possible *idle* states: *relaxed* and *prepared*. At the beginning of each motion task repetition (trial), the computer programme would pseudo-randomly choose from the two *idle* states and prompted a visual cue on the display for the human subject to follow. The *relaxed* state means the upperlimb muscles would stay relaxed and see no more muscle activation beyond what were necessary for softly holding the hand in position. On the other hand, the *prepared* state means the upperlimb muscles could be engaged but to produce reasonable isometric contraction so that the hand firmly held onto the joystick. We did not prescribe further specifications about muscle activation patterns since we would accept certain variations of the two *idle* tasks.

Each subject performed an equal number of *prepared* idle tasks and *relaxed* idle tasks. Henceforth, each subject’s data set consists of 30 repetitions of each of the four combinational tasks: *Prepared*-*Push*, *Prepared*-*Pull*, *Relaxed*-*Pull*, *Relaxed*-*Pull*.

Due to possible lapse and human errors, not every motion instance would constitute a valid trial data for our study. We have carefully compared the registered hand (joystick) motion signals with the event log from the computer programme while also assessing the sEMG quality visually. We removed those motion task trials of either apparent or suspicious incorrect task performance (e.g. no reaction after the cue). The statistics of valid trials after this data screening process are given in Table 1. The vast majority of the original trials are deemed of high quality.

There were two important time points of interest in each motion task trial: the task cue onset, and the actual motion onset. While the former can be readily extracted from the data log, the latter must be determined from the motion data (joystick position signals) instead. Specifically, the hand motion onset was marked by the first abrupt deviation of the joystick’s X position signal (linked to the pull-push motion) from the signal level during the preceding *idle*-period. Therefore, our motion onset detection algorithm used a threshold mechanism on this deviation to determined the motion onset time point, and the threshold value was empirically determined so as to reliably detect the first moment of the motion.

The time interval between the two time points is the so-called reaction time, which indicates how quickly a human subject reacts to a visual motion cue. We provide the statistics of the reaction times in Table  1. However, we notice that these reaction times are noticeably longer than conventional PVT study results. This apparent discrepancy could be attributed to two facts: firstly, our protocol is not a standard PVT study and we actually did not instruct the human subjects to react at maximum possible speed – because we were concerned with unnecessarily large motions even beyond the realm of the upperlimb-hand; secondly, the protocol used multiple and non-predictable action tasks, which would require more complex visual processing and decision making than in conventional PVT. Therefore, these ”reaction” time data are not comparable to those from conventional PVT studies.

**Table 1:** The number of valid motion trials for each *idle*-*motion task* combination (columns 2-5); and the statistics of the reaction time. As explained in the text, these ”reaction” time data should not be compared with standard PVT results.

Subj. No.	*Relax.*-*Push*	*Prep.*-*Push*	*Relax.*-*Pull*	*Prep.*-*Pull*	Reaction time *Relaxed*	Reaction time *Prepared*
1	30	25	29	27	645ms±95ms	628ms±100ms
2	25	28	30	28	666ms±96ms	600ms±68ms
3	28	27	28	27	550ms±50ms	547ms±57ms
4	27	30	28	26	684ms±71ms	698ms±125ms
5	28	26	26	26	902ms±177ms	965ms±168ms
6	26	29	28	27	666ms±177ms	635ms±130ms
7	28	22	23	25	830ms±107ms	652ms±121ms
8	29	29	29	23	483ms±74ms	420ms±63ms
9	28	26	30	28	699ms±161ms	634ms±93ms
10	28	29	28	30	762ms±112ms	611ms±80ms
11	29	29	28	29	1078ms±164ms	945ms±161ms

### The machine learning model and the intention prediction algorithm

**Figure 3:**
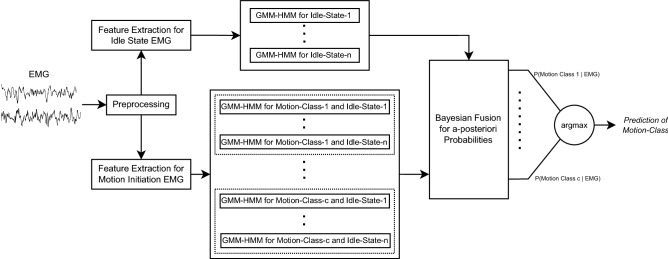
Diagram of the Hybrid Bayesian Dynamic Model for sEMG-based Motion Intention Prediction.

**Figure 4:**
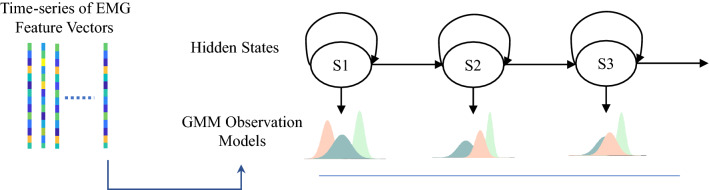
Diagram of a particular GMM-HMM for a sequence of sEMG feature vectors.

As described earlier, we aim to design and experimentally validate a hierarchical dynamic Bayesian learning network that can accurately learn and predict the continuous and complex process of motion initiation which may start from different *idle* states. The overall network design is depicted in Fig. 3 that consists of two groups of GMM-HMMs. The upper group is used to describe sEMG processes for every idle state, called idle state model (ISM). The lower group is used to describe motion initiation processes for every motion class and every idle state, called motion initiation model (MIM). The final decision can be derived from computing the *a-posterior* probability of each motion class given the idle state sEMG and the motion initiation process sEMG. Note that thanks to the dynamic Bayesian modelling, the method is able to compute the *a-posterior* probabilities at any time point during the motion initiation process, hence enabling motion prediction ahead of the motion onset.

Let the idle sEMG be $X_{idle}$. As will be detailed in “Gaussian mixture model‑hidden markov model (GMM‑HMM)” section, each GMM-HMM for idle sEMG will learn the dynamics of idle sEMG for a particular idle state. For an unknown-state idle sEMG, it will compute the *a-posterior* probability of each *idle* state, namely, *P*(*Prepared*) and *P*(*Relaxed*). Similarly, let the motion initiation sEMG be $X_{MI}$. Each GMM-HMM for motion initiation will learn the dynamics of sEMG during the motion initiation process starting from a particular idle state and ending at a particular motion onset.

For simplicity of mathematical expression and also for generalization of the method, we denote the motion intention class by $c_{mot}$ (in the present study, it is either the *Push* or the *Pull* class), and let the idle state class be $c_{idle}$ (in the present study, it is either the *Prepared* or the *Relaxed* state).

Hence, the a posterior probability of a particular motion intention for observed sEMG data is given by

1 $$\begin{aligned} P(c_{mot}|\{X_{idle},X_{MI}\}) = \sum _{c_{idle}} P(c_{mot}|X_{MI}, c_{idle}) P(c_{idle}|X_{idle}) \end{aligned}$$

The structure of GMM-HMM is illustrated in Fig. 4. Our choice of GMM-HMM for the dynamic Bayesian model of the different sEMG processes is primarily due to two reasons. GMM-HMM is known to require relatively less training data to achieve convergence. Meanwhile, the relatively high interpretability of GMM-HMM allows us to perform in-depth analyses of the motion initiation process.

### Feature extraction

**Figure 5:**
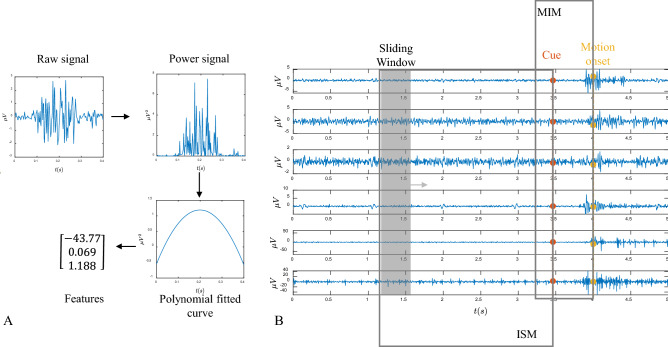
(**A**) Feature extraction by polynomial curve fitting. The selected sEMG signal segments are squared, smoothed and second-order polynomial fitted to obtain the three dimensional features. (**B**) Feature extraction for ISM and MIM. In idle state model(ISM), the signal 600ms before the cue point are used. Motion initiation model (MIM) focus on the signal from 100ms before the cue point to the motion onset point. A sliding window length of 20ms is used to extract features.

Figure 5 depicts the feature extraction method. At an arbitrary point of time, we need to convert the measured sEMG into a feature vector. The feature vector shall describe the dynamics of the sEMG activity at that time point. Practically, we can compute the sEMG activity level in a short time window. As illustrated in Fig. 5, we first compute the instantaneous power of the high-passed processed sEMG waveform and use a 2nd-order polynomial to describe the dynamics.

Without loss of generality, let the power curve be $x(\tau )$ where $\tau$ is the relative time to the present time point. The 2nd-order polynomial approximation is given by

2 $$\begin{aligned} \hat{x}(\tau )= a_0+a_1 \tau + a_2 \tau ^2 \end{aligned}$$

with the least L-2 normal error, i.e. minimal $\sum _\tau (\hat{x}(\tau )-{x}(\tau ))^2$. In this work, this approximation is readily done with the Matlab cure-fitting toolbox.

In order to enhance the computational efficiency of the system, we used principal component analysis (PCA) to reduce the dimensionality of the data. Principal component analysis is a popular method for data compression. For a giving data matrix *A* with *n* samples and *m* dimensions, after normalizing the data in every dimension to produce the normalized matrix *B* from *A*, the eigenvalues $\Lambda = [\lambda _1, \lambda _2, \lambda _3,\ldots , \lambda _m]$ and eigenvectors $W = [w_1, w_2, w_3,..., w_m]$ of the covariance matrix of *B* is calculated. Then, the first few eigenvectors $W_p = [w_1, w_2, w_3,..., w_p]$ with the largest eigenvalues will be used to transform the original data into a reduced feature vector representation. In this paper, we followed the 95% total variance rule to keep the largest eigenvectors in each instance of training an ISM/MIM (see the next subsection). The feature dimension is reduced to 3 and 4 in ISM and MIM respectively. Without loss of generality, we denote the dimension of the feature vector by *p* in the following.

### Gaussian mixture model-hidden markov model (GMM-HMM)

After extracting the feature sequences from the raw sEMG signals, the sequences will be used to build the hidden Markov model (HMM), which is a type of Bayesian classifier and has been used effectively to categorize EEG and sEMG signals^18,26,27^. HMM calculates the posterior probability of an observed sequence of features, which is expressed as a Gaussian mixture model.

The Gaussian mixture model (GMM) is a probabilistic model that assumes that all data points are generated by a finite number, denoted as *M*, of Gaussian distributions, and it is a useful technique to quantify the statistical characteristics of the features when using the hidden Markov model (HMM). Following the modeling of the feature series by the GMM, the HMM will be used build the ISM/MIM.

By using the mixture weight of each distribution ($c_m$), the mean vector ($\vec {\mu }_m$), and the Covariance matrix (${\Sigma }_m$) of each distribution, a GMM can parametrically represent an observation vector ($\vec {x}$)^28^.

3 $$\begin{aligned} P(\vec {x}) = \sum _{m=1}^{M}c_{m} \mathscr {N}(\vec {x}|\vec {\mu _{m}},{\Sigma _{m}}) \end{aligned}$$

where

4 $$\begin{aligned} \mathscr {N}(\vec {x}|\vec {\mu _{m}},{\Sigma _{m}})= & {} \frac{1}{\sqrt{(2\pi )^p|\Sigma _m|}}exp(\frac{-(\vec {x}-\vec {\mu _{m}})^T{\Sigma _{m}}^{-1}(\vec {x}-\vec {\mu _{m}})}{2}), \end{aligned}$$

5 $$\begin{aligned} \sum _{m=1}^{M}c_{m}= & {} 1. \end{aligned}$$

The Hidden Markov Model (HMM) is a widely used method for evaluating discrete time series. It is a term for a Markov process with hidden unknown states. HMM is based on two fundamental assumptions. To begin, it is assumed that the state ($S_t$) of the hidden Markov chain at any instant *t* depends only on the previous moment’s state ($S_{t-1}$), and is independent of the states and observations at other moments, as well as independent of *t*. Second, it is assumed that the observation ($O_t$) at any given time *t* is determined solely by the state at that time, regardless of other observations or the states of the Markov chain.

In this way, for the HMM’s training phase, two components, transition matrix $\textbf{A}$ and emission matrix $\textbf{B}$, should be trained. $a_{ij}$ in matrix $\textbf{A}$ means $P(S_j|S_i)$, which is the transition probability from $S_i$ to $S_j$. $b_j(O_t)$ in matrix $\textbf{B}$ means the probability of the observation $O_t$ under the state $S_j$, which can be expressed as $P(O_t|S_j)$. $O_t$ is from an observation sequence, $O = \{O_0,O_1,...,O_{T-1}\}$. In Gaussian Mixture Model-Hidden Markov Model, the emission matrix is combined by several Gaussian distributions and the expectation maximization (EM) algorithm is usually used to estimate the parameters of the model^29,30^. EM consists of two main steps. The first step is expectation (E-step), which is based on the assumed values of the parameters, calculates the expected estimates of the unknown variables. In the second step, maximization (M-step), the maximum likelihood estimates of the parameters of the model are given based on the estimates of the unknown variables as follows,

6 $$\begin{aligned} \hat{\mu }_{jm}= & {} \frac{\sum _{t =1}^{T}\gamma _t(j,m)O_t}{\sum _{t =1}^{T}\gamma _t(j,m)} \end{aligned}$$

7 $$\begin{aligned} \hat{\Sigma }_{jm}= & {} \frac{\sum _{t =1}^{T}\gamma _t(j,m)(O_t-\hat{\mu }_{jm})(O_t-\hat{\mu }_{jm})^T}{\sum _{t =1}^{T}\gamma _t(j,m)} \end{aligned}$$

8 $$\begin{aligned} \hat{c}_{jm}= & {} \frac{\sum _{t =1}^{T}\gamma _t(j,m)}{\sum _{k =1}^{M}\sum _{t =1}^{T}\gamma _t(j,k)} \end{aligned}$$

9 $$\begin{aligned} \hat{a}_{ij}= & {} \frac{\sum _{t =1}^{T}\xi _t(i,j)}{\sum _{n =1}^{N}\sum _{t =1}^{T}\xi _t(i,n)} \end{aligned}$$

where $\gamma _t(j,m) = P(s_t = j,c_t = m | O)$, $\xi _t(i,j) = P(s_t=i, s_{t+1} = j|O)$, *N* means the number of states and *T* means the length of the observation sequences.

In the testing stage, HMM is used to solve two fundamental problems. One is called *Decoding* and the other is called *Likelihood*. In the *Likelihood* problem, the trained HMM model ($\lambda = (\textbf{A},\textbf{B})$) is used to determine the likelihood of an observation sequence, $O = \{O_0,O_1,...,O_{T-1}\}$, and this problem is solved by forward algorithm. The forward algorithm calculates the observation likelihood, $P = (O|\lambda )$ , by calculating $\alpha _t(j) = P(O_0,O_1,...,O_t,s_t=j|\lambda )$ recursively,

10 $$\begin{aligned} \alpha _1(j)= & {} \pi _j b_j(O_1) \end{aligned}$$

11 $$\begin{aligned} \alpha _t(j)= & {} \sum _{i=1}^N \alpha _{t-1}(i) a_{ij} b_j(O_t) \end{aligned}$$

where $\pi _j$ is the initial probability, $a_{ij}$ is the transition probability from state *i* to state *j*, and finally $P (O|\lambda ) = \sum _{i=1}^N\alpha _{T-1}(i)$. Another issue of concern when utilizing the HMM model is the *Decoding* problem. That is not only concerned with the final classification result, but also with the process of changing the hidden state of the observed sequence. This kind of decoding problem is often solved by the Viterbi algorithm. The Viterbi algorithm is a general decoding algorithm that is based on dynamic programming for finding the shortest path of a sequence.

For an observation sequence $O = \{O_0,O_1,...,O_{T-1}\}$, the $V_{t-1}(j)$, which is the previous Viterbi path probability whose final state is *j* and from the previous time step, is given by the recurrence relation,

12 $$\begin{aligned} V_{1}(j)=\, & {} \pi _j P(O_1|j) \end{aligned}$$

13 $$\begin{aligned} V_{t}(j)= & {} \max _{j \in N } (V_{t-1}(i)a_{ij}P(O_t|j)) \end{aligned}$$

where $\pi _j$ is the initial probability, $a_{ij}$ is the transition probability from state *i* to state *j* and $V_{t}(j)$ is the probability that the first *t* observations whose final state is *j* correspond to the state sequence. Then at the end backtracing the best path to the beginning, the decoded sequence of states $S = \{ s_{0},\dots ,s_{T-1}\}$ is determined. In this paper, the Viterbi algorithm will be used for motion intention decoding. In the part of motion intention prediction, for the time-series observation sequence, we will decode and analyze the hidden state transition sequence with the highest probability.

## Experimental results

This section consists of a series of numerical experiments that examine the hypothesis and the system performance and characteristics of interest. We first examine the hypothesis that the knowledge of the idle state will allow for more accurate dynamic Bayesian modeling of the motion initiation sEMG process. This can be tested using the prediction accuracy of the proposed method but assuming the true idle state is known. We then evaluate the actual performance of the proposed GMM-HMM for idle state recognition and compare the result with that by a typical conventional static model. The study continues with the evaluation of the proposed method under the practical situation when the idle state is unknown. Lastly, we examine the evolution of the decoded hidden states of sEMG during the motion initiation process and relate that to the increasing prediction accuracy.

### Dynamic Bayesian modelling with and without the knowledge of the idle state

In this experiment, we evaluate the performance of the motion initiation models (MIMs) with the knowledge of the *idle* state and investigate the effect of the *idle* states on the accuracy. We train and test GMM-HMMs with mixing four Gaussian distributions and three hidden states. Two models are trained and tested separately based on the two *idle* states (*Prepared* and *Relaxed*). Considering the difference between the different subjects, the models are trained and tested individually. All experiments in this paper were conducted with a five-fold cross-validation to obtain reliable model performance. The performance of the model in distinguishing *Pull* and *Push* motions in two pre-intention muscle force levels is shown in the first two columns in Table 2.

**Table 2:** Performance of motion prediction in different idle states.

Subject	Accuracy(%)		Accuracy(%)	
No.	Idle-State-Dependent Method		Conventional GMM-HMM Method	
	(Relaxed)	(Prepared)	(Relaxed)	(Prepared)
1	$95.00\pm 4.56$	$84.55\pm 12.74$	$88.33\pm 7.34$	$80.54\pm 13.71$
2	$98.33\pm 3.73$	$96.36\pm 4.98$	$98.33\pm 3.73$	$92.87\pm 7.57$
3	$94.55\pm 8.13$	$87.27\pm 13.79$	$92.72\pm 7.61$	$76.36\pm 15.21$
4	$100.00\pm 0$	$100.00\pm 0$	$100\pm 0$	$96.51\pm 4.78$
5	$98.18\pm 4.06$	$98.00\pm 4.47$	$94.54\pm 8.13$	$98\pm 4.47$
6	$94.18\pm 8.85$	$94.54\pm 8.13$	$100\pm 0$	$96.36\pm 8.13$
7	$93.78\pm 9.08$	$89.56\pm 14.56$	$91.78\pm 13.09$	$83.11\pm 11.85$
8	$93.03\pm 3.91$	$78.91\pm 7.91$	$82.87\pm 5.66$	$71.27\pm 9.22$
9	$96.67\pm 4.56$	$96.18\pm 5.24$	$96.67\pm 4.56$	$98.18\pm 4.06$
10	$100\pm 0$	$80\pm 13.94$	$96.36\pm 4.97$	$88.33\pm 9.50$
11	$96.51\pm 4.77$	$87.72\pm 10.25$	$90.91\pm 15.74$	$77.42\pm 14.27$
Average	${96.38\pm 5.53}$	${90.28\pm 11.30}$	$93.86\pm 8.75$	$87.18\pm 13.14$

Accuracy is reported as $mean \pm standard \ deviation$.

Average accuracy rates are tested for statistical significance by Wilcoxon signed-rank test on the accuracy being higher with the idle-state-dependent method than with the conventional method. (*Relaxed *State$p=0.028<0.05$, *Prepared *State $p = 0.021<0.05$).

From the results, it can be seen that the model has a high accuracy for motion intention prediction. The accuracy of motion recognition in the *relaxed* state is higher than that in the *prepared* state. On average, the accuracy was $96.38\%\pm 5.53$, in the *relaxed* state, and $90.28\%\pm 11.30$ in the *prepared* state. This indicates that the accuracy of motion recognition is about six percentage points higher in the *relaxed* state than in the *prepared* state, and the smaller variance means more consistent performance.

To statistically analyze our results, we first used the Lilliefors test to figure out whether the data came from a normal distribution so that we could select an appropriate statistical test. The test results of accuracy rate data reject the null hypothesis. In other words, the accuracy rates are not normally distributed. Thus, we used Wilcoxon signed-rank test to statistically test our results. Because our experiment is conducted with a five-fold cross-validation, we make pairwise comparisons on the idle-state-dependent method and conventional method in each fold. For the idle-state-dependent method, we can also test of significance of differences in the model performances on the two *idle* states (*Prepared* and *Relaxed*).

Wilcoxon signed-rank test shows that the accuracy difference between the two *idle* states was statistically significant $(p=0.0003<0.01)$, which indicates that there is a clear difference in model performance for the two states of preparation and relaxation. It is also shown that the different *idle* states have a significant effect on motion recognition.

To further show the advantage of training different *idle* states separately, the idle state dependently trained models are compared with the GMM-HMM model trained conventionally (hereafter ‘Conventional GMM-HMM Method’). In the conventional way, the model is trained by all sEMG signals regardless *idle* states. This conventional method has been used to estimate the joint angle^20^ and hand movements^31^ successfully. The experiment uses mixed data from two *idle* states to train the GMM-HMM with four Gaussian distributions and three hidden states, and separate data from two *idle* states to measure the model’s performance in each *idle* state. The results are shown in the last two columns of the Table 2.

As can be seen from the results, the accuracy rates in the conventional method are lower than the accuracy rates in separately training method in either *relaxed* or *prepared* states. The differences between the two methods are significant $(p < 0.05)$ both in the two *idle* states. Furthermore, in five of eleven subjects, the proposed method has a better performance than the conventional method in both *idle* states. In four subjects, the proposed method gets a higher accuracy rate in one *idle* state and the accuracy in the other *idle* state is equal to the conventional method. This indicates that training the motion intention sequences but ignoring the *idle* states leads to confusion in the model.

### The performance of idle state recognition

From the above section, the results indicated that the *idle* states have an impact on motion intention recognition. So, recognition of different *idle* states benefits the whole model performance. For the recognition of *idle* states (prepared or relaxed), the signal from 600ms before the motion command (cue point) was used. For sEMG signals, Bartuzi *et al.* claims that 30-80*Hz* sEMG component depends on muscle force^32^. Thus, a linear discriminate analysis (LDA) model using frequency domain features is compared with the *idle* state model (ISM) model in this paper. In ISM, a GMM-HMM with four Gaussian distributions and three hidden states was trained with the features extracted through the method we introduced in “Feature extraction” section. The results are shown in Table 3.

**Table 3:** Comparison of the recognition performance of idle states.

Subject	Accuracy(%)	Accuracy(%)
No.	(GMM-HMM)	(LDA)
1	$79.28\pm 3.97$	$76.52\pm 8.27$
2	$75.84\pm 2.87$	$57.94\pm 10.81$
3	$98.13\pm 2.55$	$98.18\pm 4.06$
4	$54.18\pm 12.95$	$58.53\pm 15.59$
5	$98.13\pm 2.55$	$97.14\pm 4.26$
6	$88.87\pm 10.52$	$87.27\pm 6.74$
7	$57.84\pm 7.48$	$61.37\pm 10.46$
8	$99.09\pm 2.03$	$99.09\pm 2.03$
9	$72.45\pm 7.76$	$70.83\pm 8.31$
10	$83.69\pm 6.05$	$83.62\pm 5.65$
11	$73.96\pm 10.89$	$66.08\pm 10.38$
Average	${80.13\pm 16.26}$	$77.87\pm 16.44$

Accuracy is reported as $mean \pm standard \ deviation$.

From the results, it can be found that the hidden Markov model is slightly more accurate for the majority of participants than the LDA method but the difference is not significant. Among the different participants, the difference in accuracy is significant. One point worth discussing is that the model is very variable for individual subjects. For Subject 3, Subject 5, and Subject 8, both models can achieve a near 100% correct rate. In contrast, for Subject 4, the *idle* state discriminate rate in both models is around 50%, implying that the models don’t work, because in this task the chance level is 50%. Since the results of the model are good for most of the subjects, it can be inferred that the failure of the model to discriminate *idle* states for Subject 4 is not the problem with the model, but rather due to the fact that the subject did not respond well to the relaxation and preparation instructions while performing the experimental data collection. In other words, Subject 4 kept upper limb muscle groups in the same *idle* state throughout the experiment, so the *idle* state is not discriminant. This analysis can also be corroborated by Table 2. In Table 2, we can also find that for Subject 4 accuracy in both *idle* states, relaxed and prepared, is 100%. Even though training the GMM-HMM model neglected the various *idle* states, the accuracy rates are still high.

### The performance of motion intention prediction

To demonstrate the validity of the proposed hierarchical model, we compared the proposed hierarchical dynamic Bayesian model and the conventional GMM-HMM. Table 4 shows the results of the two models. From the results, we can find the proposed model performance is better in 10 of 11 subjects. For Subject 4, the proposed approach performs equally with the conventional method. The proposed approach’s average accuracy is $93.83\pm 6.41$, which is greater and more stable than the conventional method $(p = 0.0021<0.01)$.

**Table 4:** Prediction accuracy of hierarchical dynamic Bayesian model and conventional GMM-HMM.

Subject	Accuracy(%)	Accuracy(%)
No.	(Hierarchical Dynamic Bayesian Model)	(Conventional GMM-HMM Method)
1	$90.19\pm 8.38$	$84.70\pm 2.29$
2	$97.31\pm 2.45$	$94.70\pm 3.61$
3	$90.91\pm 8.50$	$77.27\pm 5.57$
4	$99.09\pm 2.03$	$99.09\pm 2.03$
5	$98.09\pm 2.61$	$90.60\pm 8.83$
6	$98.22\pm 2.43$	$96.36\pm 3.80$
7	$94.73\pm 6.44$	$91.84\pm 5.72$
8	$86.30\pm 5.76$	$81.82\pm 14.73$
9	$97.31\pm 4.04$	$94.66\pm 3.72$
10	$90.54\pm 3.51$	$89.74\pm 8.74$
11	$89.44\pm 6.12$	$86.09\pm 7.14$
Average	${93.83\pm 6.41}$	$89.71\pm 8.98$

Accuracy is reported as $mean \pm standard \ deviation$.

Average accuracy rates are tested for statistical significance by Wilcoxon signed-rank test on the accuracy being higher with the hierarchical dynamic Bayesian model than with the conventional method. ($p=0.0021<0.01$).

**Figure 6:**
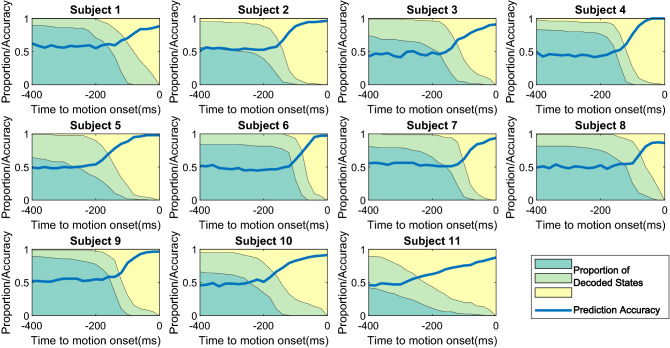
Converging hidden-states vs. prediction accuracy during motion initiation. The shaded regions of different colors represent the changing distribution of the 3 decoded hidden states among all the samples, hence always add up to 1 at each time point: the dark green region represents the first-order hidden state, the light green the second-order hidden state, and the yellow the third-order hidden state. The curve of the prediction accuracy rate is also plotted.

It is interesting to investigate the two associated processes during the motion initiation: how the hidden states converge from the idle state to the definitive motion onset time point, and how the accumulated prediction power varies at the same time. To this end, we examine each time point up to 400ms ahead of motion onset, and run the proposed motion prediction algorithm on the partial multi-sEMG series (up to that particular time point). The result is illustrated in Fig. 6.

As expected, the accuracy rate increases monotonically from the chance level of 50%. The increase is minimal, if any, when the first-order hidden state is dominant. The 2nd state seems transitional but not well defined, as there is hardly any point of time that sees the state becomes the dominance. The 3rd state indicates the definitive stage, which may come into the dominant position either abruptly (Subject 6 and Subject 7 being notable examples), or quite gradually over a long time range (Subject 11 being the notable example). Furthermore, the progress of the 3rd state into the dominant position appears to correlate well with the increasing curve of the classification accuracy in each and every case.

The graphs indicate that, generally, the prediction accuracy begins to increase noticeably at 100ms ahead of the motion onset. Subject 5 and Subject 10 are examples that this important time point can become as early as 200ms, indicating that predictive information (*Pull* vs *Push*) in multi-sEMG activation begins to form as early as 200ms.

Subject 11 shows distinctive patterns in the distribution of hidden states and in the accuracy curve. The changes are mostly gradual yet steadily all the way. At the same time, we notice that this subject performed the tasks differently from the others: as indicated in Table 1, this subject is the only one with an average reaction time beyond 1000ms. And there is also a large variance in the reaction time among the samples. This means that the motion initiation process samples from this subject are inconsistent – producing poorly defined time structure in the decoded hidden state space as well as in the prediction accuracy. Nevertheless, the decoded hidden state distribution is still well correlated with the accuracy, suggesting the proposed method is able to capture the underlying temporal structure even if not well defined in time.

## Discussions and conclusion

We have built a data collection protocol for acquiring human sEMG data associated with hand motion on a computer joystick while considering two specific *idle* states (*prepared* or *relaxed*) and a binary hand motion task (*pull* or *push*). We have integrated an sEMG device to record 6 muscle activities of 6 select muscle groups of the arm controlling the hand motion. We have run a series of offline training-prediction tests using cross-validation and examined the prediction accuracy of the proposed method in comparison with the conventional machine learning method that does not address the variability of the idle state.

Our results have shown that, as expected, the knowledge about the idle state (being *relaxed* or *prepared*) has a significant impact on the accuracy of dynamic Bayesian models for the motion initiation sEMG processes. The improvement in prediction accuracy is consistent across nearly all of the 11 human subjects in this study. It is noteworthy the prediction accuracy tends to be higher under the *relaxed* idle state. As we understand it generally, the Bayesian prediction accuracy is determined by the uncertainty (randomness) of the signal/pattern. This result seems to indicate that the designated *prepared* idle task would see a larger variability in the motion initiation sEMG process. This is related to the nature of the designated *prepared* idle task that would see a variety of variational isometric muscle contractions. The designated *relaxed* idle task, on the other hand, would not see such a variation in isometric muscle contractions. Hence, we may hypothesize that an appropriate break-down of the ‘prepared’ idle state class may lead to more accurate dynamic Bayesian modelling of the motion initiation sEMG processes.

It is also interesting to note that the result supports the use of dynamic Bayesian models for the idle state recognition than the static modelling approach. That can be attributed to the inherent variability of the sEMG processes that may exhibit temporal structures in isometric contraction (or non-contraction) activities.

Apparently, the test result depicted in Fig. 6 shows different paces of changing state distribution and of the changing accuracy rate. We hypothesise that the paces are related to how the human subjects actually perform the cued motion task. For example, the data of Subject 5, Subject 10 and Subject 11 showed long trends of increasing accuracy. This is correlated with the three being among the four subjects of the slowest average reaction time. It is likely the motion initiation process tends to be longer in those slower reaction cases. On the other hand, Subject 7 may constitute an exceptional case in which the average reaction time is among the longest, but both the converging trend of hidden states and the accuracy rate see trivial change until very near the motion onset time. A plausible explanation is that the subject might tend to start the motion initiation significantly later than others.

Note that the primary purpose of this research was to validate a new detection method designed with an experimental study. Tentatively we used a three-state configuration for the hidden states, and it will be interesting to see if a more articulated design will capture finer intricate details in the temporal structures of the multi-sEMG processes during motion initiation. Besides, the current study only considered two classes of movements. Future follow-up studies may look into more motion and idle classes.

Recent years have seen increasing interest in deep learning (DL) for decoding human motion intention especially in hand gesture classification^33,34^. Deep learning is generally used as a discriminative classifier to recognize different intentions during the gesture process or after the gesture is completed, which differs from the present subject matter which aims to construct a generative model of the motion initiation process prior to the beginning of the motion. Nonetheless, deep learning provides for automatic learning of discriminative feature extraction from a large amount of data, which is in contrast to the general machine learning methodology that depends largely on the effectiveness of feature engineering. On the other hand, DL requires a large training data set from each individual since large inter-subject variations are often present in the EMG activation processes. That can be prohibitively demanding in actual use scenarios. To address this issue, recent years have seen proposals of new DL-EMG algorithms using transfer learning^35,36^. It will be interesting to study how transfer learning DL may apply to the decoding of motion initiation processes. Furthermore, this research currently focuses on modeling the dynamic processes of EMG activities prior to motion onset, than on analyzing specific EMG features. Hence only a small basic set of EMG features were used. Future research using DL may consider SHapley Additive exPlanations or alike to understand the behaviour of the machine learning model^37^.

In conclusion, we have shown that integrated machine learning of the *idle* state can significantly improve the intention prediction of motion initiation sEMG. Particularly, we designed a hierarchical machine learning method that comprises two sets of related dynamic Bayesian networks that either learns sEMG of the *idle* states, or learns idle-state-dependent sEMG activity processes during motion initiation. We also reported an experimental study in which we built a data collection protocol for human sEMG data associated with hand motion on a computer joystick, with designated idle state tasks and a binary motion task. Our results suggest that the idle state recognition favors the dynamic Bayesian model over a static classification model. We have also shown that the proposed machine learning method can produce significantly higher accuracy in a 2-class motion intention prediction task than the conventional GMM-HMM method. The performance of our work is still restricted by the limited dataset, including the number of participants and the complexity of the movements. We intend to collect more diverse data in the future in order to extend our work to predict human movement intention. Moreover, we suggest that future research may study the *prepared* idle state and explore more sophisticated models especially for the last two hidden states in the present dynamic Bayesian models.

## Data Availability

The datasets used and analyzed in the present work are available from the corresponding author upon proper request.
